# Hospital Festivals and Meetings

**Published:** 1895-05-04

**Authors:** 


### HOSPITAL FESTIVALS AND MEETINGS.

### THE FRENCH HOSPITAL.

The annual dinner in aid of the funds of the French Hospital and Dispensary took place on Saturday, April 27th, at the Hotel Metropole. The French Ambassador (Baron de Courcel) presided, and many friends of the Institution were present, amongst them the Italian Ambassador, Alderman Sir Stuart Knill, M. C. D. Bourcart, Comte de Pontavice de Heussey, Dr. Louis Vintras, and Sir William MacCormac. The French Ambassador was received with loud cheers on rising to propose the toast of "The Queen, the Prince and Princess of Wales, and the other members of the Royal Family," and referred in happy terms to the sympathy extended by the Queen towards the country of France. In proposing "The Founders and Benefactors of the French Hospital and Dispensary" special mention was made of the interest ever taken in the institution by M. Waddington, and a grateful tribute paid to the energy and zeal displayed in its behalf by Dr. Vintras, one of its founders, and one who had ministered most to its needs. Sir Stuart Knill, in proposing "The Health of the Chairman," said the banquet represented a union of charity, all nations joining together in promoting the welfare of a hospital which was for the benefit of all. During the evening subscriptions and donations amounting to £2,600 were announced, which included £25 from M. de Courcel.

